# Whole‐exome sequencing of breast cancer, malignant peripheral nerve sheath tumor and neurofibroma from a patient with neurofibromatosis type 1

**DOI:** 10.1002/cam4.551

**Published:** 2015-10-03

**Authors:** John Richard McPherson, Choon‐Kiat Ong, Cedric Chuan‐Young Ng, Vikneswari Rajasegaran, Hong‐Lee Heng, Willie Shun‐Shing Yu, Benita Kiat‐Tee Tan, Preetha Madhukumar, Melissa Ching‐Ching Teo, Joanne Ngeow, Aye‐Aye Thike, Steven George Rozen, Puay‐Hoon Tan, Ann Siew‐Gek Lee, Bin‐Tean Teh, Yoon‐Sim Yap

**Affiliations:** ^1^ Division of Neuroscience and Behavioral DisordersDuke‐National University of Singapore Graduate Medical SchoolSingapore169857Singapore; ^2^ Lymphoma Genomic Translational Research LaboratoryDivision of Medical OncologyNational Cancer Centre Singapore11 Hospital DriveSingapore169610Singapore; ^3^ Laboratory of Cancer EpigenomeDivision of Medical SciencesNational Cancer Centre Singapore11 Hospital DriveSingapore169610Singapore; ^4^ Department of General SurgerySingapore General HospitalOutram RoadSingapore169608Singapore; ^5^ Division of Surgical OncologyNational Cancer Centre Singapore11 Hospital DriveSingapore169610Singapore; ^6^ Division of Medical OncologyNational Cancer Centre Singapore11 Hospital DriveSingapore169610Singapore; ^7^ Department of PathologySingapore General Hospital20 College RoadAcademia, Level 7, Diagnostics TowerSingapore169856Singapore; ^8^ Laboratory of Molecular OncologyDivision of Medical SciencesNational Cancer Centre SingaporeSingaporeSingapore; ^9^ Office of Clinical & Academic Faculty AffairsDuke‐National University of Singapore Graduate Medical SchoolSingaporeSingapore; ^10^ Department of PhysiologyYong Loo Lin School of MedicineNational University of SingaporeSingaporeSingapore; ^11^ Laboratory of Cancer TherapeuticsDivision of Cancer and Stem Cell BiologyDuke‐National University of Singapore Graduate Medical School8 College RoadSingapore169857Singapore; ^12^ Laboratory of Chromatin RegulationCancer Science Institute of Singapore14 Medical DriveSingapore117599Singapore; ^13^ Faculty of Health SciencesSchool of MedicineUniversity of AdelaideAdelaideSouth AustraliaAustralia

**Keywords:** Breast cancer, neurofibromatosis type 1

## Abstract

Neurofibromatosis type 1 (NF1) is a genetic disorder characterized by the development of multiple neurofibromas, cafe‐au‐lait spots, and Lisch nodules. Individuals with NF1 are at increased risk of developing various tumors, such as malignant peripheral nerve sheath tumor (MPNST), pheochromocytoma, leukemia, glioma, rhabdomyosarcoma, and breast cancer. Here, we describe the exome sequencing of breast cancer, MPNST, and neurofibroma from a patient with NF1. We identified a germline mutation in the *NF1* gene which resulted in conversion of leucine to proline at amino acid position 847. In addition, we showed independent somatic *NF1* mutations in all the three tumors (frameshift insertion in breast cancer (p.A985fs), missense mutation in MPNST (p.G23R**)**, and inframe deletion in dermal neurofibroma (p.L1876del‐Inf)), indicating that a second hit in *NF1* resulting in the loss of function could be important for tumor formation. Each tumor had a distinct genomic profile with mutually exclusive mutations in different genes. Copy number analysis revealed multiple copy number alterations in the breast cancer and the MPNST, but not the benign neurofibroma. Germline loss of chromosome 6q22.33, which harbors two potential tumor suppressor genes, *PTPRK* and *LAMA2*, was also identified; this may increase tumor predisposition further. In the background of NF1 syndrome, although second‐hit *NF1* mutation is critical in tumorigenesis, different additional mutations are required to drive the formation of different tumors.

## Introduction

Neurofibromatosis type 1 (NF1) is a relatively common genetic disorder characterized by the development of multiple neurofibromas, cafe‐au‐lait spots, and Lisch nodules, with estimated incidence of 1 in 2,000 to 1 in 5,000 individuals worldwide 1. The *NF1* gene on chromosome 17q11.2 is a classic tumor suppressor gene. Its product, neurofibromin is an important negative regulator of the Ras cellular proliferation pathway 2, 3. Individuals with NF1 are at increased risk of developing various tumors, including MPNST, pheochromocytoma, leukemia, glioma, and rhabdomyosarcoma 4. More recently, an increased risk of breast cancer has also been reported 5, 6.

The mechanism of pathogenesis of NF1‐associated breast cancer remains unknown; limited data suggest an aggressive biology of breast cancer in NF1 patients 7, 8. Previously, studies on genetic aberrations in MPNST focused on only a limited set of genes, reporting mutations in *TP53* and second hit *NF1*, multiple copy number alterations, and deletion of *CDKN2A* 4, 9, 10, 11. Recently published studies now report frequent somatic aberrations in *EED* and *SUZ12* as well, both of which are chromatin‐modifying genes 12, 13, 14.

In this study, we sequenced the exomes of breast cancer, MPNST, dermal neurofibroma, and matched whole‐blood from a single NF1 patient. The objectives were to unravel the genomic complexity of different neoplastic manifestations of NF1 and to identify somatic mutations that potentially drive these tumors.

## Materials and Methods

### Patient

The subject of this study fulfilled NIH Consensus Development Conference diagnostic criteria for NF1; family history was also positive for NF1. At the age of 39 years, she was diagnosed with right breast cancer. Histopathological examination revealed a 40 mm grade 3 invasive ductal carcinoma with metastasis to 1 of 17 lymph nodes. Estrogen‐ and progesterone‐receptor status was positive. HER2 was 2+ in 30% of the cells by immunohistochemistry, and borderline positive on fluorescent in situ hybridization (FISH) testing with a ratio of HER2 to chromosome 17 signals from 60 nuclei scored as 2.2, and an average of 4.7 HER2 signals per nucleus. Adjuvant chemotherapy as well as trastuzumab and tamoxifen were administered postoperatively, in addition to radiotherapy.

Three years later, she presented with a rapidly growing soft tissue mass beneath the right buttock. Excision of this mass and a separate dermal neurofibroma on the right buttock was performed; the pathological diagnosis for the mass was malignant peripheral nerve sheath tumor arising from a plexiform neurofibroma. Sections showed a hypercellular spindle cell tumor with large areas of necrosis and hemorrhage and high mitotic activity.

Blood and fresh frozen tumor specimens (breast cancer, MPNST, dermal neurofibroma) were obtained from this patient. The study was approved by the local Institutional Review Board. All the tumor specimens contained at least 70% tumor by routine histologic review with hematoxylin and eosin staining.

### Exome capture and high throughput sequencing

Three micrograms of DNA per sample were sheared using a Covaris S1 Ultrasonicator (Covaris, MA). Adaptor‐ligated libraries were constructed using Paired‐End DNA kits (Illumina, CA). Exome capture was performed using SureSelect Human All Exon Kit v3 (Agilent Technology, CA). Each sample was sequenced on two lanes of an Illumina GA‐IIx sequencer using 76‐bp paired‐end reads. The image analysis and base calling were performed using the Illumina pipeline (v1.6) with default settings.

### Sequence mapping and coverage computation

Alignment of the sequenced reads was to human reference genome hg19, using the Burrows‐Wheeler Aligner software 15. PCR duplicates were removed using SAMTools. Variants were called using a pipeline based on the Genome Analysis Toolkit (GATK) software (Broad Institute, Massachusetts). Base quality scores were recalibrated and the sequences near microindels were realigned. Consensus calling for SNVs and microindels was done with the GATK Unified Genotyper. Only well‐mapped reads and reads with fewer than four mismatches in a 40 base‐pair window were considered.

The putative SNVs and microindels were annotated against dbSNP 135 and 1000 Genomes to remove common polymorphisms, excluding cancer‐associated positions (based on presence in the COSMIC database [Wellcome Trust Sanger Institute, Cambridge, United Kingdom]). Using transcripts from the CCDS, RefSeq, Ensembl, and UCSC databases, we identified nonsynonymous mutations and classified them as tumor‐somatic if the matched normal sample had sufficient coverage to show that the variant was not present in the germline. Putative mutations were validated by Sanger sequencing.

### Copy number variation analysis

Analysis of copy number and regions of loss‐of‐heterozygosity was performed on the exome sequencing data using the ASCAT algorithm 2.0 (Allele‐Specific Copy number Analysis of Tumors) (http://heim.ifi.uio.no/bioinf/Projects/ASCAT/). For higher resolution, the blood, dermal neurofibroma and primary breast tumor samples were assayed using the Affymetrix CytoScan HD platform (Affymetrix, Inc, Santa Clara, CA). There was insufficient DNA from the frozen specimen of MPNST for the Cytoscan copy number analysis. Analysis of the array data was performed with the Chromosome Analysis Suite software (version 2.0.1) from Affymetrix.

## Results

### Whole‐exome sequencing of breast cancer, MPNST, and dermal neurofibroma in NF1 patient identified independent *NF1* mutations

Our target enrichment and sequencing achieved a mean coverage of 75, with an average of 83% of bases covered by at least 20 reads in each sample (Table S1). To identify the possible *NF1* germline mutations in this patient, we inspected all variants detected in the patient's blood DNA, and identified a heterozygous missense mutation of thymidine to cytosine (g.chr17:29,556,173). This mutation resulted in a conversion of leucine to proline at amino acid position 847, which is also present in all the tumor samples confirmed by Sanger sequencing, indicating germline mutation (Fig. S1).

**Figure 1:**
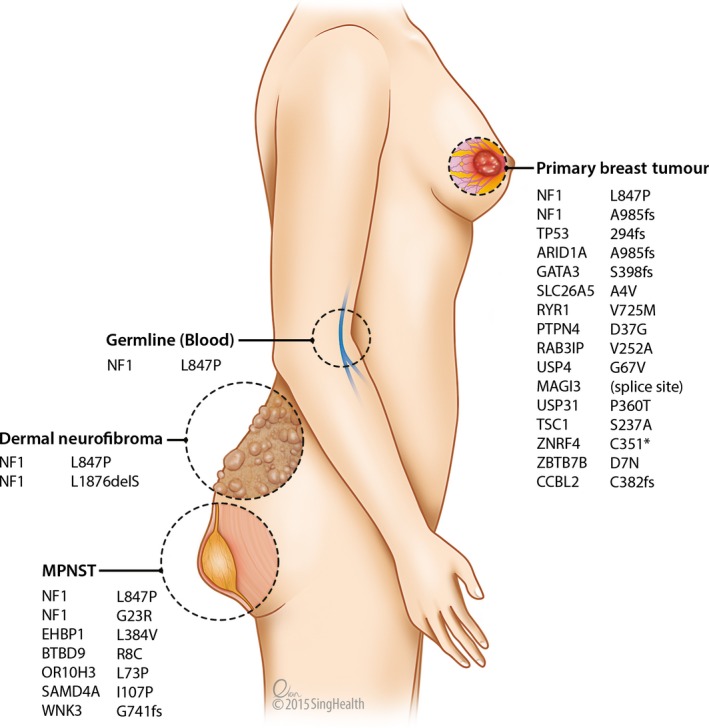
Somatic mutations in the three sequenced tumors.

**Figure 2:**
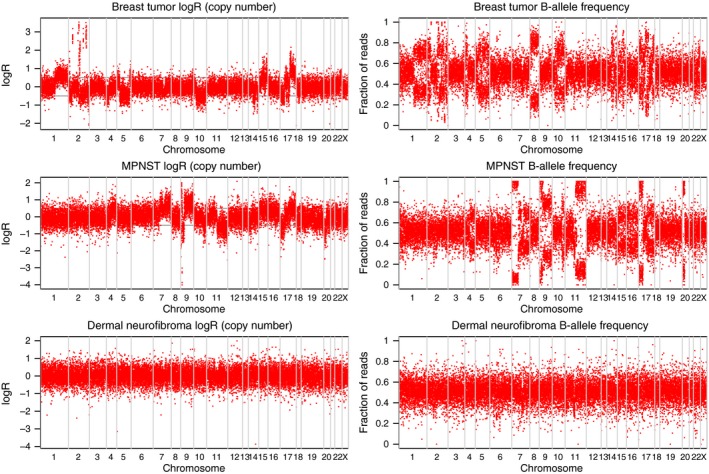
Analysis of copy number alterations in breast cancer (A), malignant peripheral nerve sheath tumor (MPNST) (B) and dermal neurofibroma (C), based on paired tumor and nontumor DNA analysis of exome sequencing data. Each red dot represents a genomic coordinate that was heterozygous in the germline sample. The breast cancer shows the most chromosomal rearrangements while the dermal neurofibroma shows none. Regions of allelic imbalance that also show a decrease in Log R typically represent Loss‐of‐heterozygosity (LOH); regions of allelic imbalance with no change in Log R show Copy‐Number Neutral LOH, and regions of allelic imbalance that correspond with increased Log R correspond to either focal amplifications (for example, several loci in chromosome 2 of the breast cancer) or large‐scale amplifications. The changes in Log R and allele frequency observed across multiple chromosomes in breast cancer and MPNST indicate allelic imbalance or copy number alterations in these samples, but not in the dermal neurofibroma.

All reported mutations were validated by Sanger sequencing in the four samples (blood, breast cancer, MPNST, and dermal neurofibroma; Fig. 1, Table S2), except the insertion of cytosine in the *NF1* gene (g.chr17:29,553,477) in the breast cancer, most probably due to repetitive sequences in that region leading to slippages in Sanger sequencing. However, close inspection using the Integrated Genome Viewer software suggests a true insertion event (Fig. 2). The somatic mutations identified in each sample were unique to the tumor, suggesting that each different type of tumor arose independently in the patient.

Consistent with the fact that malignant tumors harbor more somatic mutations, the breast cancer and MPNST harbored 15 and 6 somatic mutations respectively (Fig. 1, Table S2). In contrast, only one somatic mutation was detected in the dermal neurofibroma, a benign tumor (Fig. 1). Interestingly, this in‐frame deletion of three nucleotides resulted in a loss of leucine at amino acid position 1876 in the *NF1* gene (Table S2). Different somatic *NF1* mutations (frameshift insertion in breast cancer (p.A985fs), missense mutation in MPNST (p.G23R**)**, and inframe deletion in dermal neurofibroma (p.L1876del‐Inf)) were detected in all three tumors investigated; suggesting the importance of second‐hit aberration in *NF1* for tumorigenesis.

Several important and interesting mutations were identified in the two malignant tumors. The breast tumor harbored somatic mutations in *TP53*,* GATA3,* and *ARID1A*, which are commonly mutated or lost in breast cancer (Fig. 1). Mutations in other cancer‐associated genes such as *MAGI3*,* TSC1*,* PTPN4*,* RAB3IP,* and *RYR1* were also identified. Aberrant protein degradation may be important in this cancer, as three of the 14 mutated genes (*USP4*,* USP31,* and *ZNRF4*) in the breast cancer are involved in the ubiquitin–proteasome pathway. In the MPNST, aside from the *NF1* mutation, other mutations in cancer‐associated genes such as *EHBP1* and *WNK3* were identified.

### Extensive copy number alterations detected in breast cancer and MPNST

To determine the extent of chromosomal aberrations in the breast cancer, the MPNST, and the dermal neurofibroma, we subjected the sequence data to our in‐house modified ASCAT analysis. Extensive chromosomal aberrations were observed in the breast cancer and the MPNST samples. Unlike the profiles seen in the breast cancer and MPNST, the benign dermal neurofibroma genome is “silent”, indicating that the neurofibroma genome is highly stable (Fig. 2).

Gains and losses in several cancer‐related genes were identified in the breast cancer and MPNST samples through ASCAT analysis (Table S3). Consistent with a previous report on chromosomal aberrations in MPNST, losses were more common than gains in the MPNST on ASCAT analysis 16. Homozygous losses were found in *CDKN2A* and *CDKN2B*, which play important roles in cell cycle control, and in *ARID4B*, a chromatin remodeling gene. Heterozygous losses were observed in *TP53* and *EED*, a component of the Polycomb repressive complex 2 (PRC2), which is reported to be frequently altered in a recent exome sequencing study on MPNSTs 12.

Potential oncogenes amplified in the breast cancer include *GREB1*,* NRXN1*,* MGAT5, PKP4*,* DAPL1*,* ITGB6,* and *RBMS1*, which may be implicated in carcinogenesis, proliferation, and invasion. A focal amplification of around 3 Mb in chromosome 2, containing the *FXBO11* and *MSH6* genes, was estimated to contain over 20 copies of the affected genes. We observed heterozygous loss of several cancer‐associated genes, including the *CTNNA1* (catenin (cadherin‐associated protein), alpha 1) and *APC* tumor suppressor genes in the breast cancer. With the available DNA, we further confirmed our findings in the breast cancer and dermal neurofibroma using the Cytoscan platform from Affymetrix.

**Figure 3:**
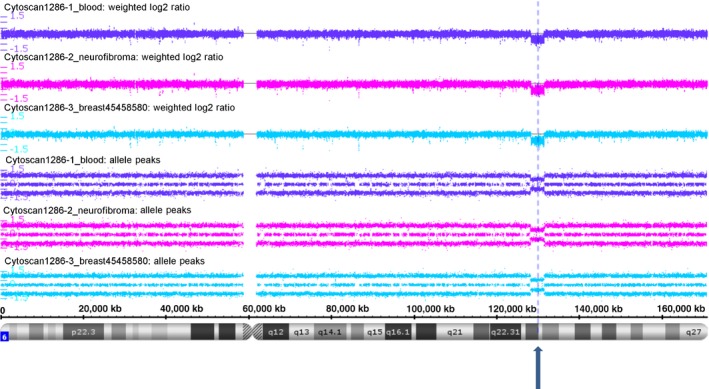
Cytoscan HD SNP Array analysis reveals a germline Loss‐of‐heterozygosity event of around 3.3 Mbases at chr6q22.33 (indicated by dotted line) which harbor *PTPRK* and *LAMA2*, in blood, breast cancer, and dermal neurofibroma.

Using Cytoscan, copies gained were observed in a large region of chromosome 2 (p25.1, p24.2, p21, q14.2, q21.2, q23.3, q24.3, q33.1, q36.1, q36.2, q36.3, and q37.2), chromosome 4 (p15.2), chromosome 15 (q22.2 and 24.2), and chromosome 17 (q12 and q21.32), whereas loss of heterozygosity was observed in chromosomes 2, 4, 5, 10, 14, 17, and 18 in the breast cancer genome (Fig. 3 and Table S4).

Interestingly, we have identified loss of chromosome 6q22.33 (Fig. 3) and gain of chromosome 14q32.33 in the DNA from the patient's blood, breast cancer, and dermal neurofibroma suggesting germline chromosomal aberrations. Two putative tumor suppressor genes, *PTPRK* and *LAMA2,* are among the 10 genes identified in the loss region of chromosome 6; these germline chromosomal alterations might predispose the patient to cancer.

## Discussion

Neurofibromatosis type 1 is a common genetic disorder, but until recently there has been a lack of data on the comprehensive mutation landscape of NF1‐associated tumors. This is set to change with the increasing availability of next generation sequencing. In fact, there is revival of interest in the *NF1* gene as somatic *NF1* aberrations are increasingly detected in sporadic tumors from individuals without neurofibromatosis type 1 syndrome 8.

Whole‐exome sequencing of the blood, breast cancer, MPNST, and dermal neurofibroma from this patient has provided invaluable insight into the somatic anomalies in this tumor predisposition syndrome. A striking feature is the finding of second‐hit *NF1* mutation at different sites of this gene in all the tumors sequenced, indicating that second‐hit mutation of this tumor suppressor gene may be a critical event in pathogenesis. *NF1* aberrations can potentially lead to activation of the Ras, MAP kinase, and PI3K‐mTOR pathways, resulting in proliferation of tumor cells 3, 17. This is consistent with the findings of a previous study which reported different somatic *NF1* alterations in multiple benign neurofibromas and a MPNST obtained postmortem from an NF1 patient 18. Denaturing high‐performance liquid chromatography (dHPLC), microsatellite analysis using RFLP markers and multiplex ligation probe amplification (MLPA) for the *NF1* gene were performed in that study 18. With whole‐exome sequencing, we have found that each tumor from the same individual also has a distinct set of genes mutated. The different clonal origins indicate that each tumor arises from independent somatic events in the background of germline *NF1* mutation.

Incidentally, we have also discovered germline loss of two potential tumor suppressor genes—*PTPRK* and *LAMA2—*on chromosome 6 (q22.33). *PTPRK* appears to be a negative regulator of adhesion, invasion, migration, and proliferation in various tumor types, including breast and colorectal cancers, gliomas, lymphoma, and melanoma cells. It may play a role in inhibition of Akt, EGFR, and beta‐catenin signaling 19, 20, 21, 22, 23. *LAMA2* encodes an extracellular matrix protein. Reduced *LAMA2* expression in hepatocellular carcinomas has been linked to a proliferative signature with poorer survival outcomes; hypermethylation of *LAMA2* has also been reported in colorectal carcinomas 24, 25. To our knowledge, germline loss of *PTPRK* and *LAMA2* has not been reported; this may increase the tumor predisposition further.

The mutation landscape of breast cancer is highly heterogeneous. In this case of NF1‐associated breast cancer, mutations in the *TP53*,* TSC1,* and *MAGI3* (PI3K/Akt/mTOR pathway) tumor suppressors are likely to cooperate with *NF1* in carcinogenesis. In addition, alterations of genes critical in other pathways, such as *ARID1A* (SWI/SNF chromatin remodeling), *GATA3* (differentiation of luminal breast cells), *PTPN4* (antiapoptosis), *RYR1* (control of cellular proliferation), and *USP4, USP31, ZNRF4* (ubiquitin–proteasome pathway) may be implicated in pathogenesis. The multiple copy number alterations detected also result in genomic instability. Gains in some of the genes involved in invasive/migratory properties (*MGAT5, PKP4, ITGB6*), cell cycle progression and regulation of apoptosis (*RBMS1*), and angiogenesis (*NRXN1*) may be involved in tumor development and progression. Amplification of *GREB1*, an early response gene in the estrogen receptor‐regulated pathway, may play a role in this estrogen receptor positive tumor.

The mechanism of pathogenesis for the MPNST is likely related to second‐hit inactivation of *NF1*, together with mutations in other genes, such as *WNK3* which plays a role in the increase of cell survival in a caspase‐3‐dependent pathway, and *EHBP1* which has been implicated in endocytic trafficking. A single‐nucleotide polymorphism in *EHBP1* has been associated with an aggressive form of prostate cancer; it may be implicated in carcinogenesis or cell survival 26. Somatic loss of critical tumor suppressors including *CDKN2A*,* CDKN2B*,* TP53,* and *EED* are also likely to contribute to tumorigenesis. Aberrations in *EED* and *SUZ12* occur frequently in sporadic, NF1‐associated and radiotherapy‐associated MPNSTs 12, 13, 14. EED and SUZ12 are core subunits of PRC2; the resulting PRC2 inactivation can lead to loss of trimethylation at lysine 27 of histone H3 (H3K27me3), and increased H3K27 acetylation which recruits bromodomain proteins and transcription factors to promote tumor growth 12, 14.

The low mutation burden and lack of copy number changes in the dermal neurofibroma is consistent with the benign nature of this tumor. Each neurofibroma consists of a heterogeneous collection of hyperproliferative Schwann cells, as well as fibroblasts, perineural cells, and mast cells 18. Although the second‐hit somatic *NF1* mutation in the Schwann cells may trigger the formation of a neurofibroma in an *NF1*‐haploinsufficient microenvironment, the precise molecular interactions among the different cells in the development of neurofibromas remain poorly understood.

Challenges remain in the detection of aberrations in the *NF1* gene. *NF1* is one of the largest human genes, with 60 exons and 350kB of genomic DNA. While next generation sequencing platforms may facilitate the simultaneous sequencing of the various exons, it may not capture intronic splicing mutations; analysis to detect aberrations and validation of mutations can pose a major challenge. Design of primers to validate the mutations detected with next generation sequencing can be complicated and not feasible at certain sites along the large gene, due to the presence of multiple pseudogenes in the human genome 8.

In summary, second‐hit inactivation of *NF1* appears to be a common feature of various tumors in the NF1 syndrome. However, the complexity of the pathogenesis of various tumors remains to be elucidated. Besides loss of *NF1* function, additional aberrations in other important cancer‐related genes involving various pathways lead to the development of specific tumors. Further investigations on additional tumor specimens from more NF1 patients will improve our understanding of the mechanisms of pathogenesis in this tumor predisposition syndrome.

## Conflict of Interest

None declared.

## Supporting information


**Figure S1.** Sanger sequencing confirms germline *NF1* variant present in all samples.

**Figure S2.** The sequencing reads as shown in the Integrated Genome Viewer software shows an insertion present (denoted with pink ‡ mark) in the breast cancer that is not present in the germline (blood) sample.

**Figure S3.** Both exome sequencing and Cytoscan HD SNP Array show LOH in chr17p for the breast cancer (both platforms) and the MPNST (only exome sequencing available).

**Table S1.** Summary of exome sequencing of the four samples.

**Table S2.** Details of the mutations detected in the exome sequencing.

**Table S3.** The estimated copy number for various cancer‐related genes, based on comparing the read‐depth coverage of the tumor sample to the matched nonmalignant sample.

**Table S4.** The estimated copy number for genes with gains or losses as detected by the Chromosome Analysis Suite software from Affymetrix for Cytoscan HD Array data.

## References

[1] Rasmussen, S. A. , and J. M. Friedman . 2000 NF1 gene and neurofibromatosis 1. Am. J. Epidemiol. 151:33–40. 10625171 10.1093/oxfordjournals.aje.a010118

[2] Cawthon, R. M. , R. Weiss , G. F. Xu , D. Viskochil , M. Culver , J. Stevens , et al. 1990 A major segment of the neurofibromatosis type 1 gene: cDNA sequence, genomic structure, and point mutations. Cell 62:193–201. 2114220 10.1016/0092-8674(90)90253-b

[3] Xu, G. F. , P. O'Connell , D. Viskochil , R. Cawthon , M. Robertson , M. Culver , et al. 1990 The neurofibromatosis type 1 gene encodes a protein related to GAP. Cell 62:599–608. 2116237 10.1016/0092-8674(90)90024-9

[4] Brems, H. , E. Beert , T. de Ravel , and E. Legius . 2009 Mechanisms in the pathogenesis of malignant tumours in neurofibromatosis type 1. Lancet Oncol. 10:508–515. 19410195 10.1016/S1470-2045(09)70033-6

[5] Sharif, S. , A. Moran , S. M. Huson , R. Iddenden , A. Shenton , E. Howard , et al. 2007 Women with neurofibromatosis 1 are at a moderately increased risk of developing breast cancer and should be considered for early screening. J. Med. Genet. 44:481–484. 17369502 10.1136/jmg.2007.049346 PMC2597938

[6] Wang, X. , A. M. Levin , S. E. Smolinski , F. D. Vigneau , N. K. Levin , and M. A. Tainsky . 2012 Breast cancer and other neoplasms in women with neurofibromatosis type 1: a retrospective review of cases in the Detroit metropolitan area. Am. J. Med. Genet. A 158A:3061–3064. 22965642 10.1002/ajmg.a.35560 PMC3505236

[7] Teh, B. T. , G. Birrell , A. Farrell , J. H. Leonard , M. K. Walters , J. M. Palmer , et al. 1997 Breast cancer in six women with neurofibromatosis type 1. Breast 6:155–160.

[8] Yap, Y. S. , J. R. McPherson , C. K. Ong , S. G. Rozen , B. T. Teh , A. S. Lee , et al. 2014 The NF1 gene revisited ‐ from bench to bedside. Oncotarget 5:5873–5892. 25026295 10.18632/oncotarget.2194 PMC4171599

[9] Nielsen, G. P. , A. O. Stemmer‐Rachamimov , Y. Ino , M. B. Moller , A. E. Rosenberg , and D. N. Louis . 1999 Malignant transformation of neurofibromas in neurofibromatosis 1 is associated with CDKN2A/p16 inactivation. Am. J. Pathol. 155:1879–1884. 10595918 10.1016/S0002-9440(10)65507-1 PMC1866954

[10] Glover, T. W. , C. K. Stein , E. Legius , L. B. Andersen , A. Brereton , and S. Johnson . 1991 Molecular and cytogenetic analysis of tumors in von Recklinghausen neurofibromatosis. Genes Chromosom. Cancer 3:62–70. 1906341 10.1002/gcc.2870030111

[11] Legius, E. , H. Dierick , R. Wu , B. K. Hall , P. Marynen , J. J. Cassiman , et al. 1994 TP53 mutations are frequent in malignant NF1 tumors. Genes Chromosom. Cancer 10:250–255. 7522538 10.1002/gcc.2870100405

[12] Lee, W. , S. Teckie , T. Wiesner , L. Ran , C. N. Prieto Granada , M. Lin , et al. 2014 PRC2 is recurrently inactivated through EED or SUZ12 loss in malignant peripheral nerve sheath tumors. Nat Genet. 46:1227–1232. 25240281 10.1038/ng.3095 PMC4249650

[13] Zhang, M. , Y. Wang , S. Jones , M. Sausen , K. McMahon , R. Sharma , et al. 2014 Somatic mutations of SUZ12 in malignant peripheral nerve sheath tumors. Nat. Genet. 46:1170–1172. 25305755 10.1038/ng.3116 PMC4383254

[14] De Raedt, T. , E. Beert , E. Pasmant , A. Luscan , H. Brems , N. Ortonne , et al. 2014 PRC2 loss amplifies Ras‐driven transcription and confers sensitivity to BRD4‐based therapies. Nature 514:247–251. 25119042 10.1038/nature13561

[15] Li, H. , and R. Durbin . 2009 Fast and accurate short read alignment with Burrows‐Wheeler Transform. Bioinformatics 25:1754–1760. 19451168 10.1093/bioinformatics/btp324 PMC2705234

[16] Bridge, R. S. Jr , J. A. Bridge , J. R. Neff , S. Naumann , P. Althof , and L. A. Bruch . 2004 Recurrent chromosomal imbalances and structurally abnormal breakpoints within complex karyotypes of malignant peripheral nerve sheath tumour and malignant triton tumour: a cytogenetic and molecular cytogenetic study. J. Clin. Pathol. 57:1172–1178. 15509679 10.1136/jcp.2004.019026 PMC1770473

[17] Johannessen, C. M. , E. E. Reczek , M. F. James , H. Brems , E. Legius , and K. Cichowski . 2005 The NF1 tumor suppressor critically regulates TSC2 and mTOR. Proc. Natl Acad. Sci. USA 102:8573–8578. 15937108 10.1073/pnas.0503224102 PMC1142482

[18] Spurlock, G. , S. Griffiths , J. Uff , and M. Upadhyaya . 2007 Somatic alterations of the NF1 gene in an NF1 individual with multiple benign tumours (internal and external) and malignant tumour types. Fam. Cancer 6:463–471. 17551851 10.1007/s10689-007-9149-5

[19] Shimozato, O. , M. Waraya , K. Nakashima , H. Souda , N. Takiguchi , H. Yamamoto , et al. 2015 Receptor‐type protein tyrosine phosphatase kappa directly dephosphorylates CD133 and regulates downstream AKT activation. Oncogene 34:1949–1960. 24882578 10.1038/onc.2014.141

[20] Agarwal, S. , M. S. Al‐Keilani , M. A. Alqudah , Z. A. Sibenaller , T. C. Ryken , and M. Assem . 2013 Tumor derived mutations of protein tyrosine phosphatase receptor type k affect its function and alter sensitivity to chemotherapeutics in glioma. PLoS ONE 8:e62852. 23696788 10.1371/journal.pone.0062852 PMC3656086

[21] Sun, P. H. , L. Ye , M. D. Mason , and W. G. Jiang . 2013 Protein tyrosine phosphatase kappa (PTPRK) is a negative regulator of adhesion and invasion of breast cancer cells, and associates with poor prognosis of breast cancer. J. Cancer Res. Clin. Oncol. 139:1129–1139. 23552869 10.1007/s00432-013-1421-5 PMC11824379

[22] Flavell, J. R. , K. R. Baumforth , V. H. Wood , G. L. Davies , W. Wei , G. M. Reynolds , et al. 2008 Down‐regulation of the TGF‐beta target gene, PTPRK, by the Epstein‐Barr virus encoded EBNA1 contributes to the growth and survival of Hodgkin lymphoma cells. Blood 111:292–301. 17720884 10.1182/blood-2006-11-059881

[23] Novellino, L. , A. De Filippo , P. Deho , F. Perrone , S. Pilotti , G. Parmiani , et al. 2008 PTPRK negatively regulates transcriptional activity of wild type and mutated oncogenic beta‐catenin and affects membrane distribution of beta‐catenin/E‐cadherin complexes in cancer cells. Cell. Signal. 20:872–883. 18276111 10.1016/j.cellsig.2007.12.024

[24] Jhunjhunwala, S. , Z. Jiang , E. W. Stawiski , F. Gnad , J. Liu , O. Mayba , et al. 2014 Diverse modes of genomic alterations in hepatocellular carcinoma. Genome Biol. 15:436. 25159915 10.1186/s13059-014-0436-9 PMC4189592

[25] Lee, S. , T. Oh , H. Chung , S. Rha , C. Kim , Y. Moon , et al. 2012 Identification of GABRA1 and LAMA2 as new DNA methylation markers in colorectal cancer. Int. J. Oncol. 40:889–898. 22038115 10.3892/ijo.2011.1245

[26] Ghalali, A. , F. Wiklund , H. Zheng , U. Stenius , and J. Hogberg . 2014 Atorvastatin prevents ATP‐driven invasiveness via P2X7 and EHBP1 signaling in PTEN‐expressing prostate cancer cells. Carcinogenesis 35:1547–1555. 24451147 10.1093/carcin/bgu019

