# Supplementary figures and images for: Whole‐exome sequencing of breast cancer, malignant peripheral nerve sheath tumor and neurofibroma from a patient with neurofibromatosis type 1
Source: Cancer Med. 2015 Oct 3;4(12):1871–8. doi: 10.1002/cam4.551 (PMC5123784; doi:10.1002/cam4.551)

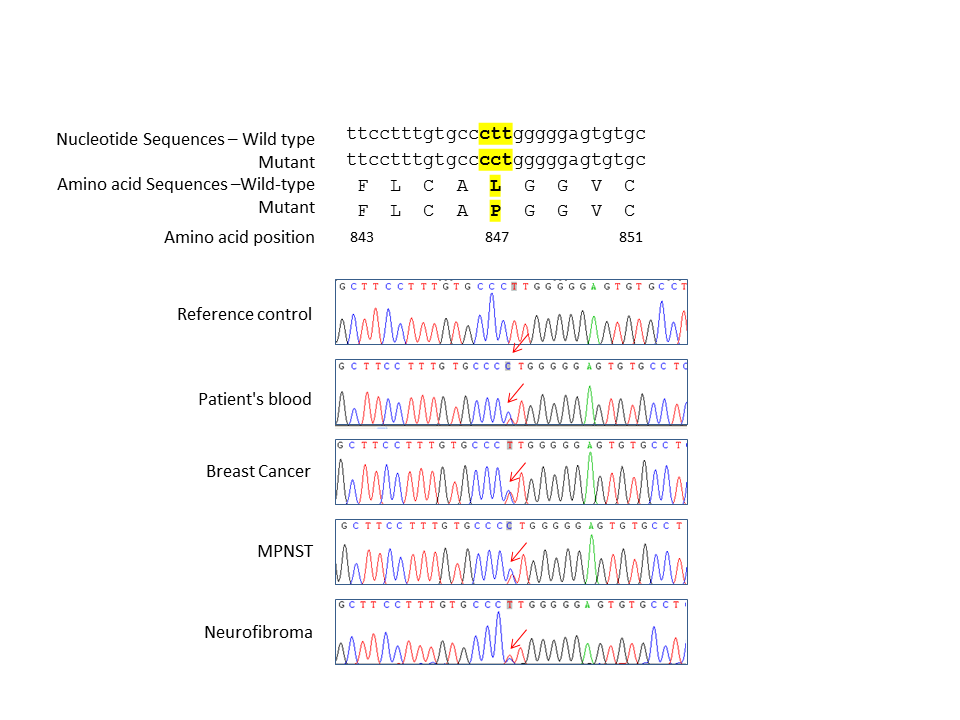

Supplement: Supplementary file 1 — Figure S1. Sanger sequencing confirms germline NF1 variant present in all samples. [file CAM4-4-1871-s001.tif]

NF1 (g.chr17:29,553,477) - p.P678Pfs

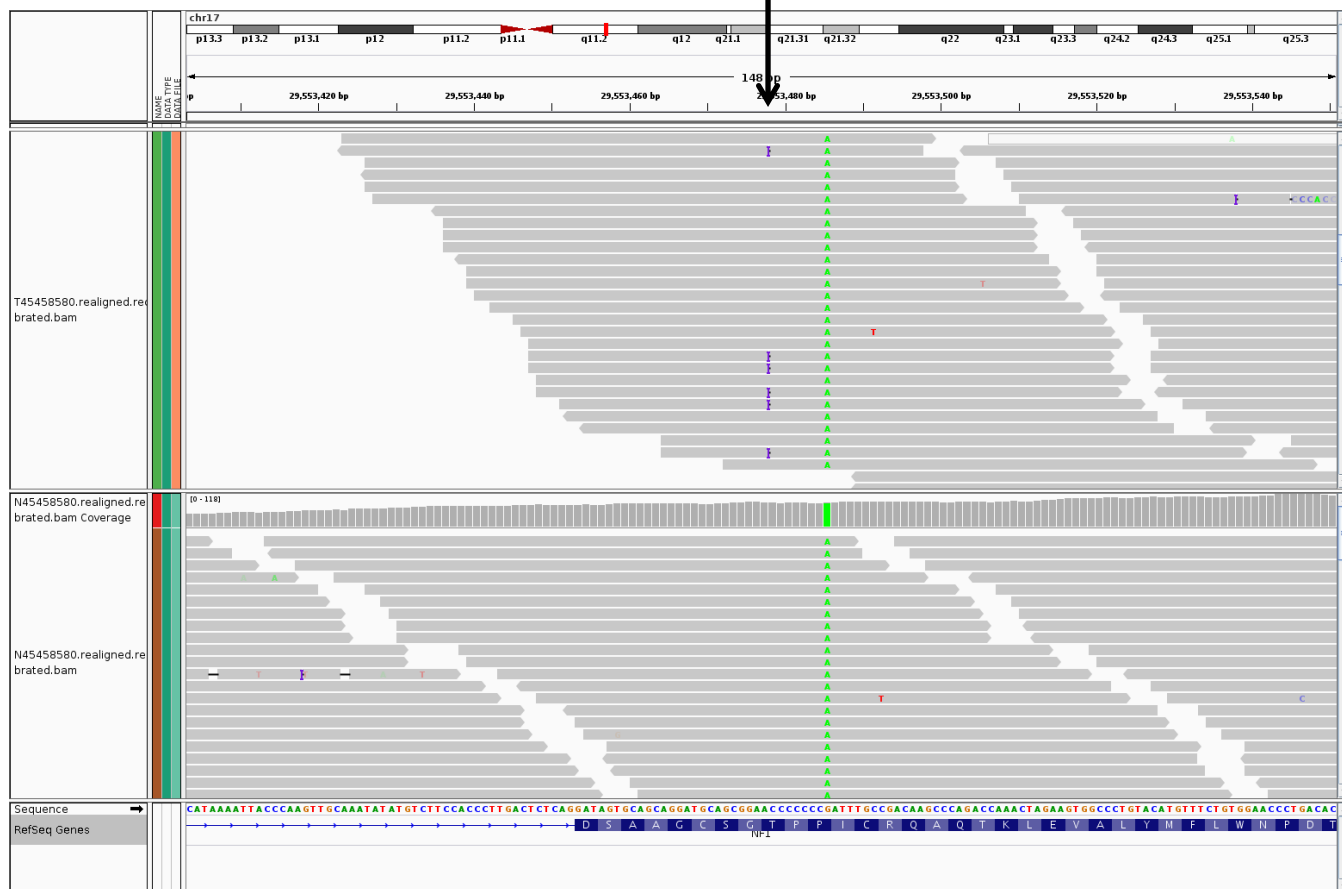

Supplement: Supplementary file 2 — Figure S2. The sequencing reads as shown in the Integrated Genome Viewer software shows an insertion present (denoted with pink ‡ mark) in the breast cancer that is not present in the germline (blood) sample. [file CAM4-4-1871-s002.pdf]

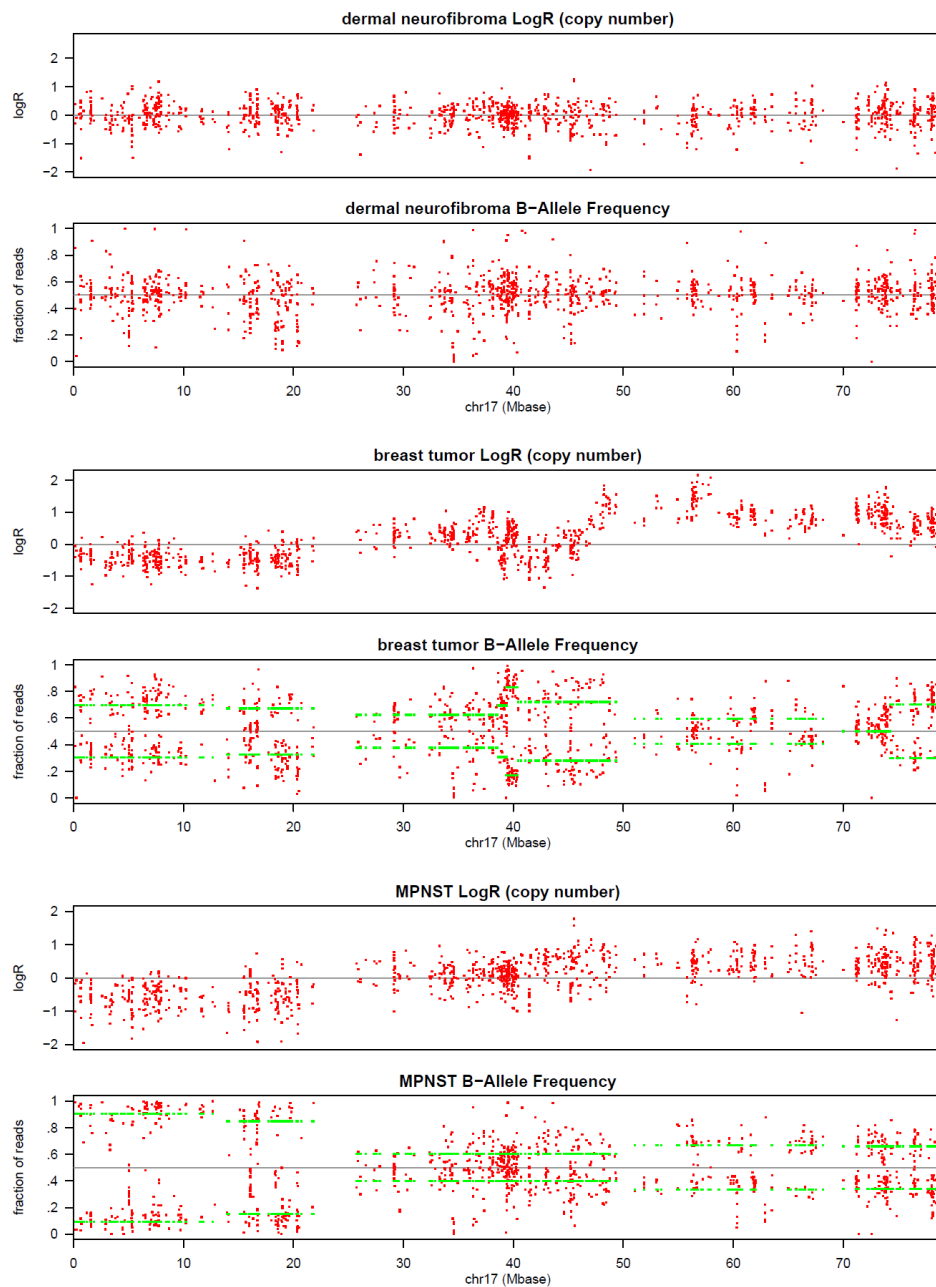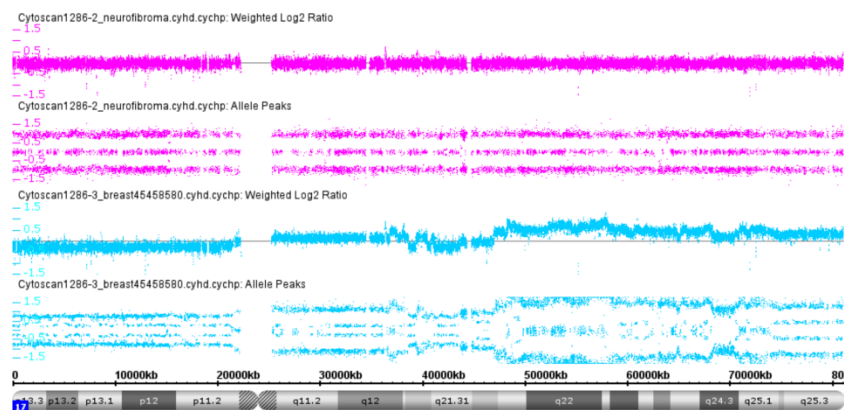

Supplement: Supplementary file 3 — Figure S3. Both exome sequencing and Cytoscan HD SNP Array show LOH in chr17p for the breast cancer (both platforms) and the MPNST (only exome sequencing available). [file CAM4-4-1871-s003.pdf]
